# Posterior Inferior Cerebellar Artery Aneurysm Presenting With Severe Vertigo and Altered Sensorium

**DOI:** 10.7759/cureus.60869

**Published:** 2024-05-22

**Authors:** Vijayashree S Gokhale, Sindhuri Goud Nimmala, Rahul Arkar

**Affiliations:** 1 Department of General Medicine, Dr. D. Y. Patil Medical College, Dr D. Y. Patil University, Pune, IND; 2 Department of Interventional Radiology, Dr. D. Y. Patil Medical College, Dr D. Y. Patil University, Pune, IND

**Keywords:** altered sensorium, vertigo, endovascular coiling, posterior inferior cerebellar artery, aneurysm

## Abstract

Posterior inferior cerebellar artery (PICA) aneurysms are relatively uncommon among intracranial aneurysms and present unique challenges due to their complex anatomical origins. PICA aneurysms arise from the vertebral artery (VA), basilar artery, or anterior inferior cerebellar artery and can have complex anatomical sites and structures. A 31-year-old female known case of trigeminal neuralgia, currently asymptomatic for the same, experienced acute vertigo, headache, and altered sensorium. On the basis of the magnetic resonance imaging of the brain with angiography, she was diagnosed with a PICA aneurysm, necessitating immediate intervention. The patient subsequently underwent endovascular coiling of the aneurysm. The successful management of this unusual case emphasizes the significance of prompt diagnosis and early intervention in managing posterior inferior cerebellar artery aneurysms, leading to a favourable outcome. The patient is on regular follow-ups and has satisfactory progress.

## Introduction

The intradural vertebral artery (VA) has an important branch, the posterior inferior cerebellar artery (PICA). This particular artery is one of the three primary arteries that supply blood to the cerebellum. PICA usually originates from the VA or sometimes from the basilar artery. PICA typically arises from the vertebral artery at an average distance of around 16 to 17 mm below the vertebrobasilar junction. The PICA provides blood to the inferior vermis, medulla oblongata, choroid plexus, tela choroidea, and fourth ventricle [1].

An aneurysm is a prominent bulge harbouring blood beneath the outer layer of an artery. The hallmark of aneurysms is that they will eventually enlarge; when this happens, there is an increased risk that they may rupture and cause grave internal bleeding or haemorrhage. PICA aneurysms are exceptionally uncommon, accounting for about 0.5% of all intracranial aneurysms [2]. A patient may become fatally ill as a result of the numerous complications that develop. Patients with PICA aneurysms have a poorer prognosis compared to those with aneurysms in other locations, given the high incidence of lower cranial nerve dysfunction in PICA aneurysm patients and their anatomical relationship with vital structures [3].

Our patient, a young female diagnosed with a posterior inferior cerebellar artery aneurysm, was treated with endovascular coiling promptly. She is now on regular follow-up with computed tomography brain and cerebral digital subtraction angiography at one, four, and six months with a stable occlusion of a previously coiled left PICA aneurysm.

## Case presentation

A 31-year-old female presented with complaints of headache, vomiting, and giddiness for a period of 20 days. There have been no complaints of fever, cough, shortness of breath, chest pain, palpitations, or seizures during this time. Vitals on arrival: pulse rate was 66 beats per minute, blood pressure was 130/80 mm Hg, oxygen saturation was 98% on room air, and respiratory rate was 18 breaths per minute. On arrival at the emergency department, the patient was drowsy and unable to respond to commands. There is no neck stiffness or rigidity. The cardiovascular and respiratory systems were normal. However, after being stabilized, the patient showed improvement in sensorium.

Lab investigations: haemoglobin was 10.2 g/dl, total leucocyte count was 7000/mm^3^, platelet count was 95,000/mm^3^. Liver and kidney function tests were within normal limits. Serum electrolytes were within the normal range. The ECG showed a sinus rhythm with a regular rate of 64 beats per minute, and the chest X-ray was normal. The 2D echo was normal. The USG abdomen and pelvis showed no abnormality.

**Figure 1:**
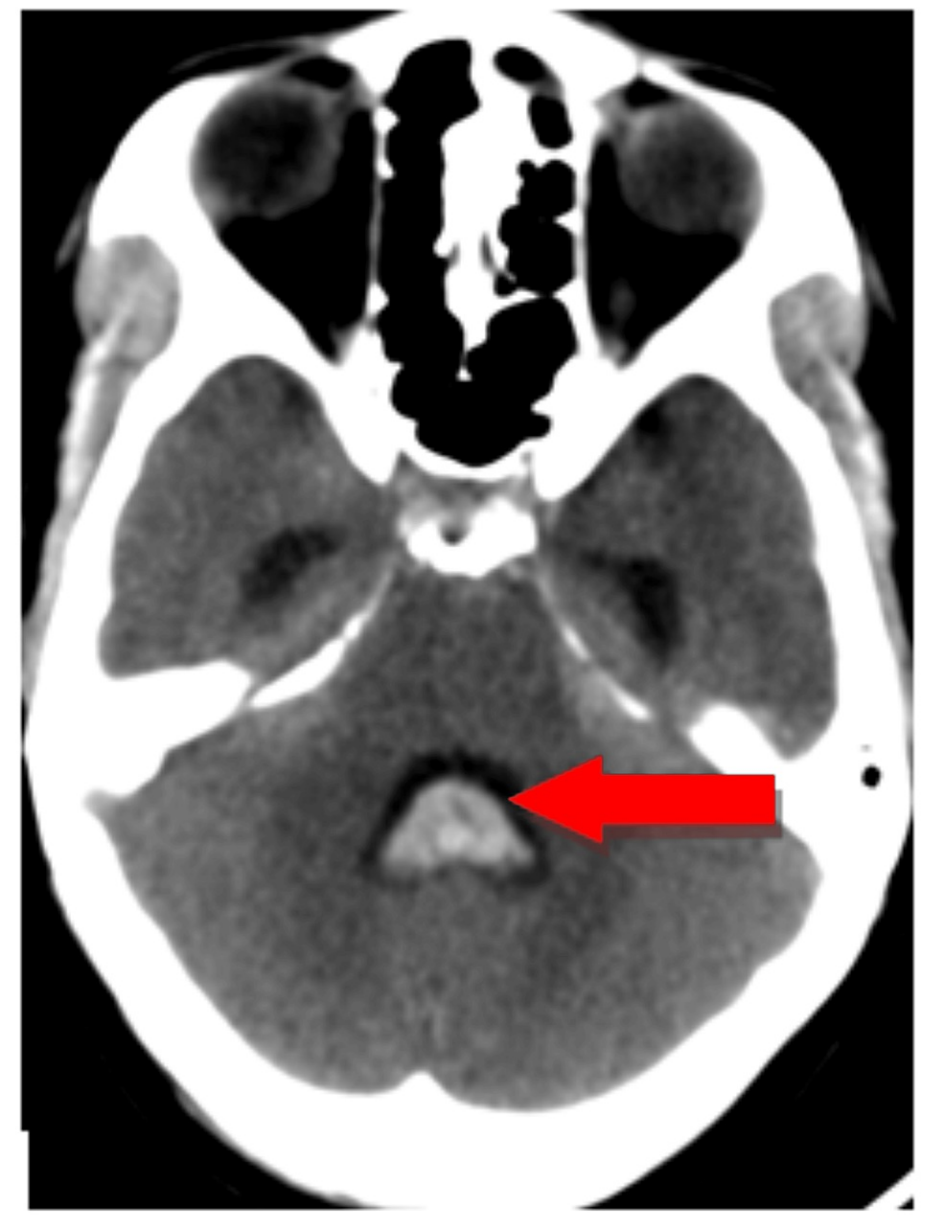
Axial non-contrast CT brain showing haemorrhage in fourth ventricle (red arrow) with extension into posterior subarachnoid space causing obstructive hydrocephalus.

On neuroimaging, "the CT brain shows intraventricular haemorrhage extending into the posterior cervical subarachnoid space, leading to obstructive hydrocephalus, as shown in Figure 1." Magnetic resonance imaging of the brain and neck angiography and venography was suggestive of acute intraventricular haemorrhage with extensions, acute subarachnoid haemorrhage with extensions, focal dilation of the left vertebral artery at the level of the foramen magnum, and obstructive hydrocephalous. A saccular aneurysm is located 4 mm away from the origin of the PICA and arises from the foetal origin of the posterior cerebral artery (PCA).

**Figure 2:**
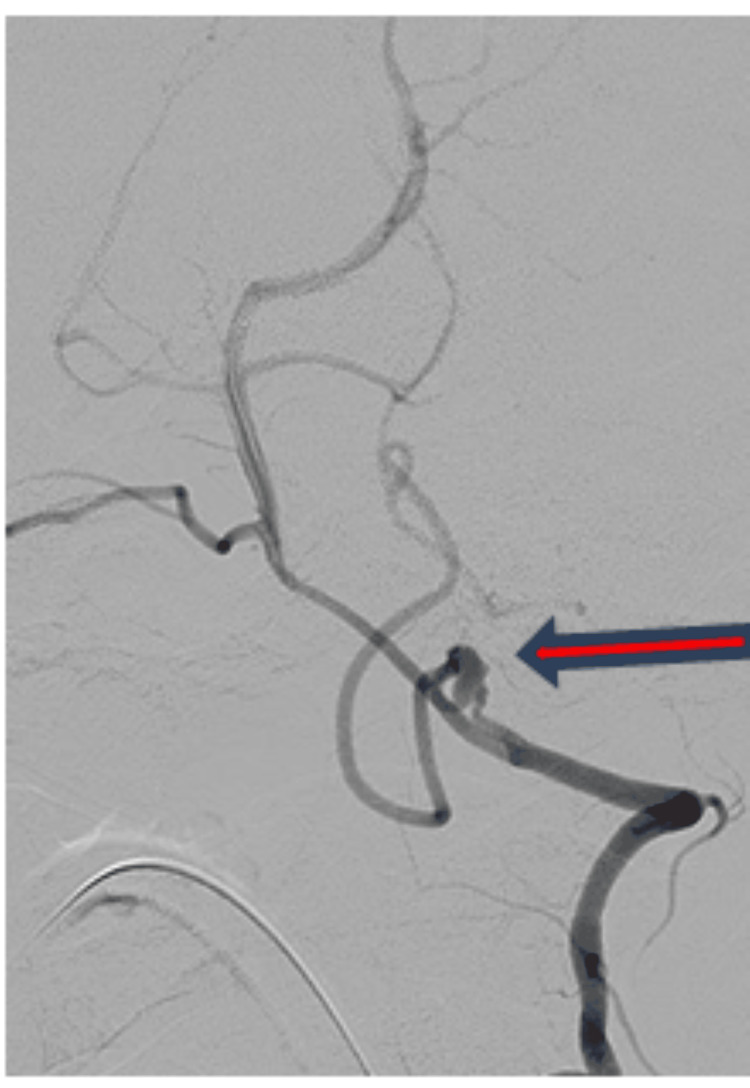
Left vertebral angiogram shows left posterior inferior cerebellar artery aneurysm (red arrow).

**Figure 3:**
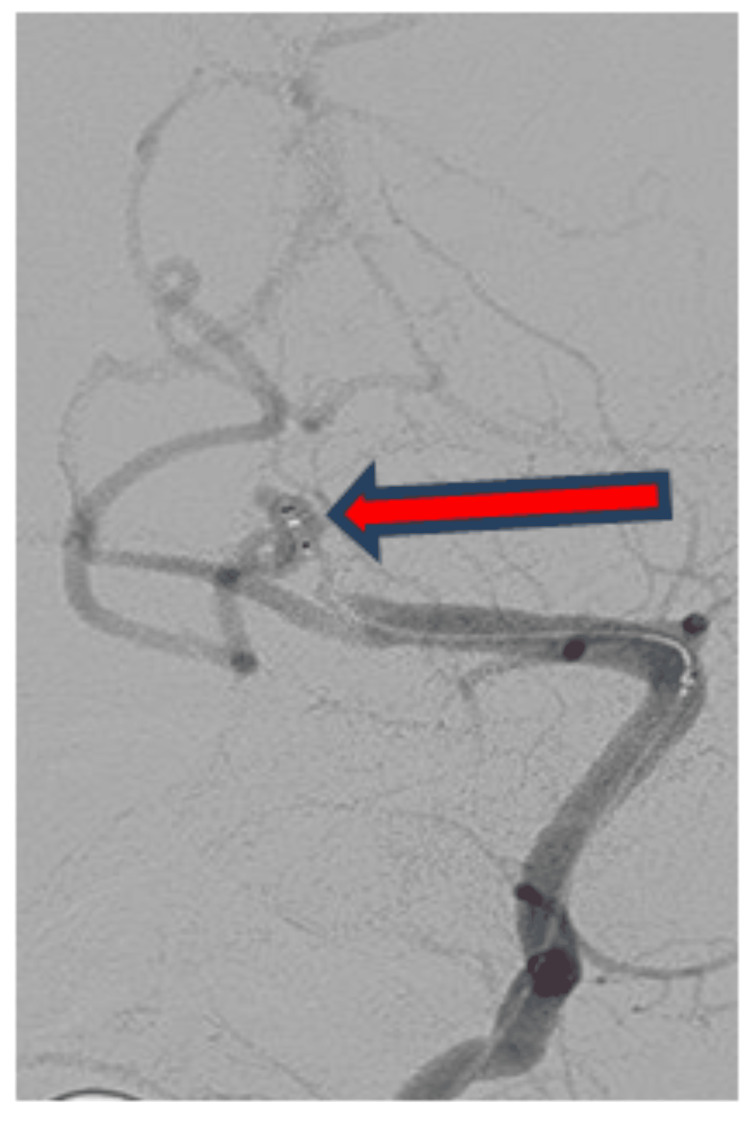
Intra-procedural angiogram shows coiling microcatheter tip (red arrow) inside left posterior inferior cerebellar artery aneurysm.

**Figure 4:**
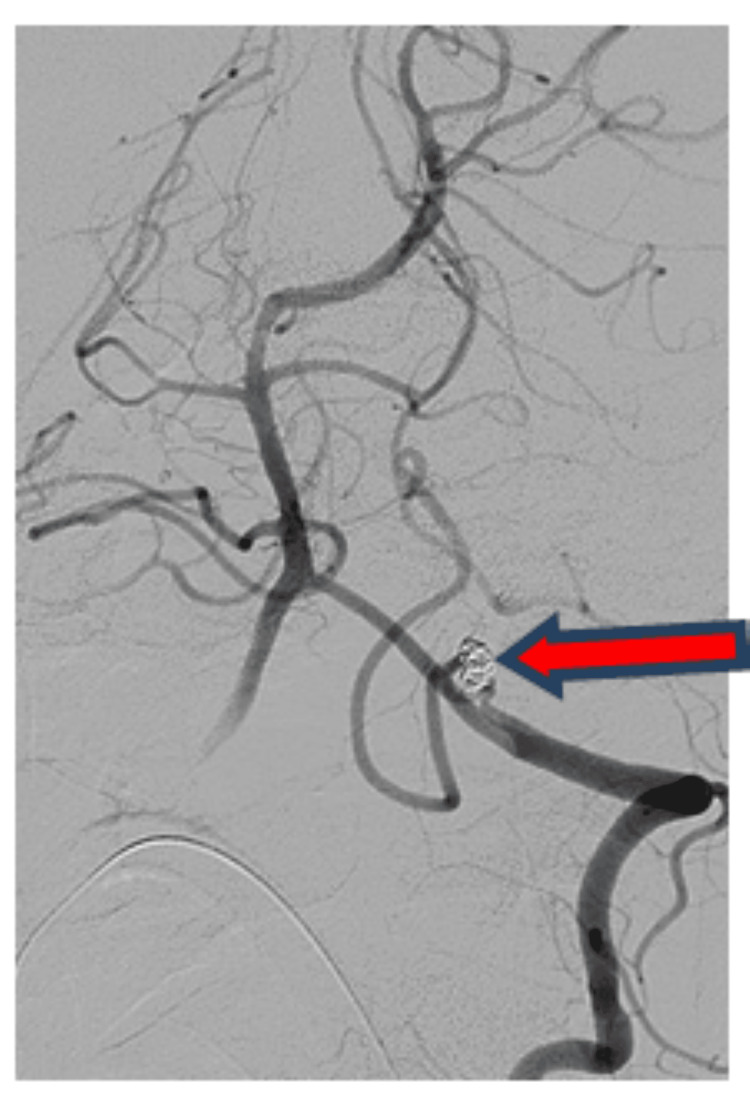
Post-coiling angiogram shows occlusion of left posterior inferior cerebellar artery aneurysm with coil mass (red arrow) with normal opacification of posterior inferior cerebellar artery and vertebro-basilar circulation.

"Prior to endovascular coiling, a digital subtraction angiography was performed, which showed a small 5.3 mm × 2.9 mm postero-supero-medially projecting, wide neck aneurysm at the lateral medullary segment of the left PICA, as depicted in Figure 2." The patient was taken for endovascular coiling for a left PICA aneurysm under general anaesthesia with an 8F right and 6F left femoral arterial access. A 6F Neuron Max long sheath was placed in the left subclavian artery, and a 5F NAVIEN guiding catheter was navigated into a distal segment of the vertebral artery. Echelon 10 microcatheter over Chikai Microwire was navigated into the PICA aneurysm. The angiogram showed no change in angiomorphology as compared to a diagnostic angiogram. "Figure 3 shows the intra-procedural angiogram of the microcatheter coiling tip (red arrow) inside the left PICA aneurysm." Subsequently, multiple detachable coils were deployed into the PICA aneurysm sac. "Post-coiling bilateral vertebral angiograms showed stable occlusion of the left PICA aneurysm with a patent basilar artery and the left posterior cerebral artery, as shown in Figure 4." The procedure was uneventful. Aneurysm showed a small daughter sac along its fundus, indicative of recently ruptured status.

The patient received cerebral decongestant injections of Mannitol and Glycerin with antiepileptics. Enoxaparin subcutaneous 0.6 ml once a day; tablet Nimodipine 60 mg every six hours; tablet Aspirin 75 mg once a day; tablet Atorvastatin 20 mg once a day in the night; tablet Betahistine 16 mg thrice daily for vertigo tapered over a few days; and antiemetics for recurrent vomiting episodes post-surgery.

Post-coiling on the fifth day, the patient developed early signs of papilledema and was treated with a tablet Acetazolamide 250 mg twice a day, which tapered over a few days.

**Figure 5:**
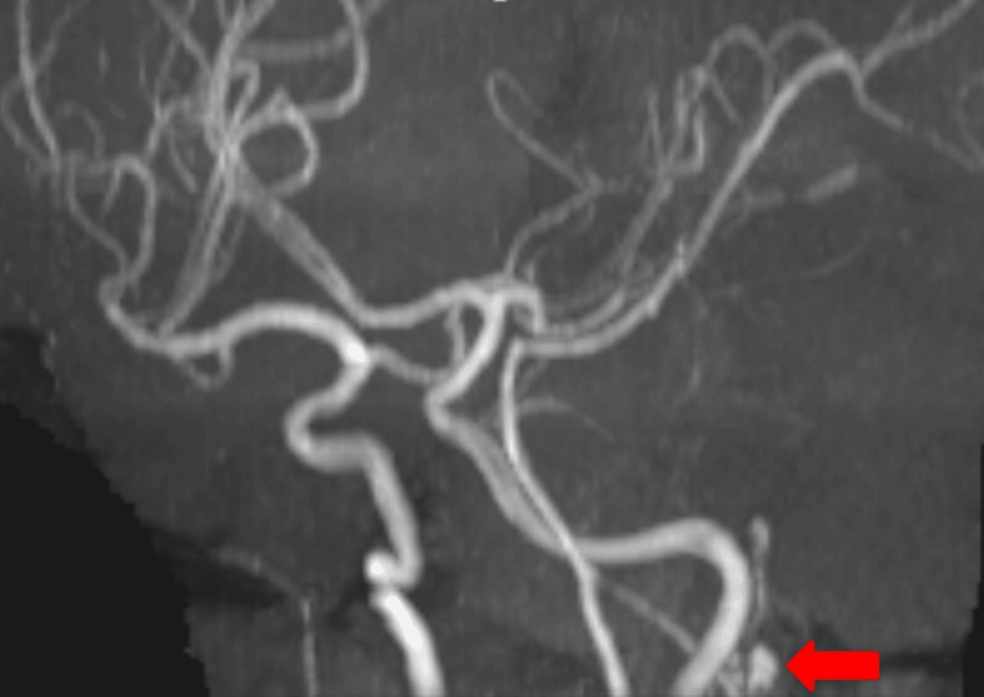
Magnetic resonance angiography brain showing left posterior inferior cerebellar artery aneurysm.

**Figure 6:**
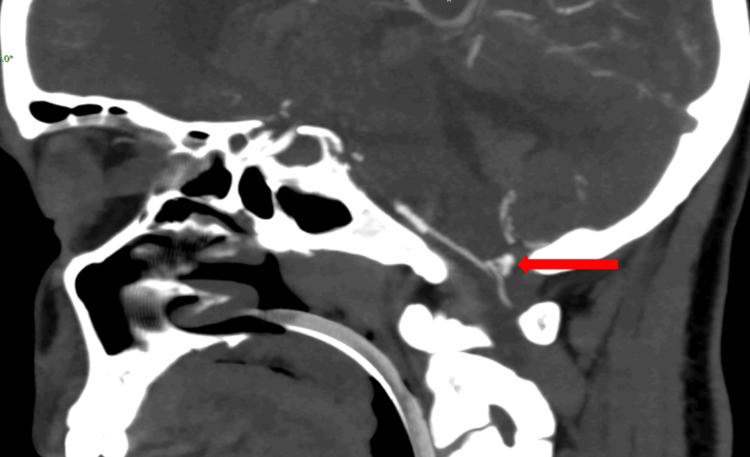
Computed tomography angiography brain showing left posterior inferior cerebellar artery aneurysm.

Magnetic resonance angiography and computed tomography angiography of the brain suggested a left PICA aneurysm, as shown in Figures 5-6, respectively.

Computed tomography of the brain plain post-coiling was repeated on day 6, which showed metallic streaks in the region of the left PICA aneurysmal coil implant with intraventricular haemorrhage and subarachnoid haemorrhage. MRI brain screening with neck angiography post-coiling on day 15 revealed minimal residual IV bleeds and hemosiderin deposits in the left ventricle; no aneurysm was seen. The patient's condition improved, her vertigo and vomiting subsided, she gained the ability to walk with support, and the hospital discharged her.

**Figure 7:**
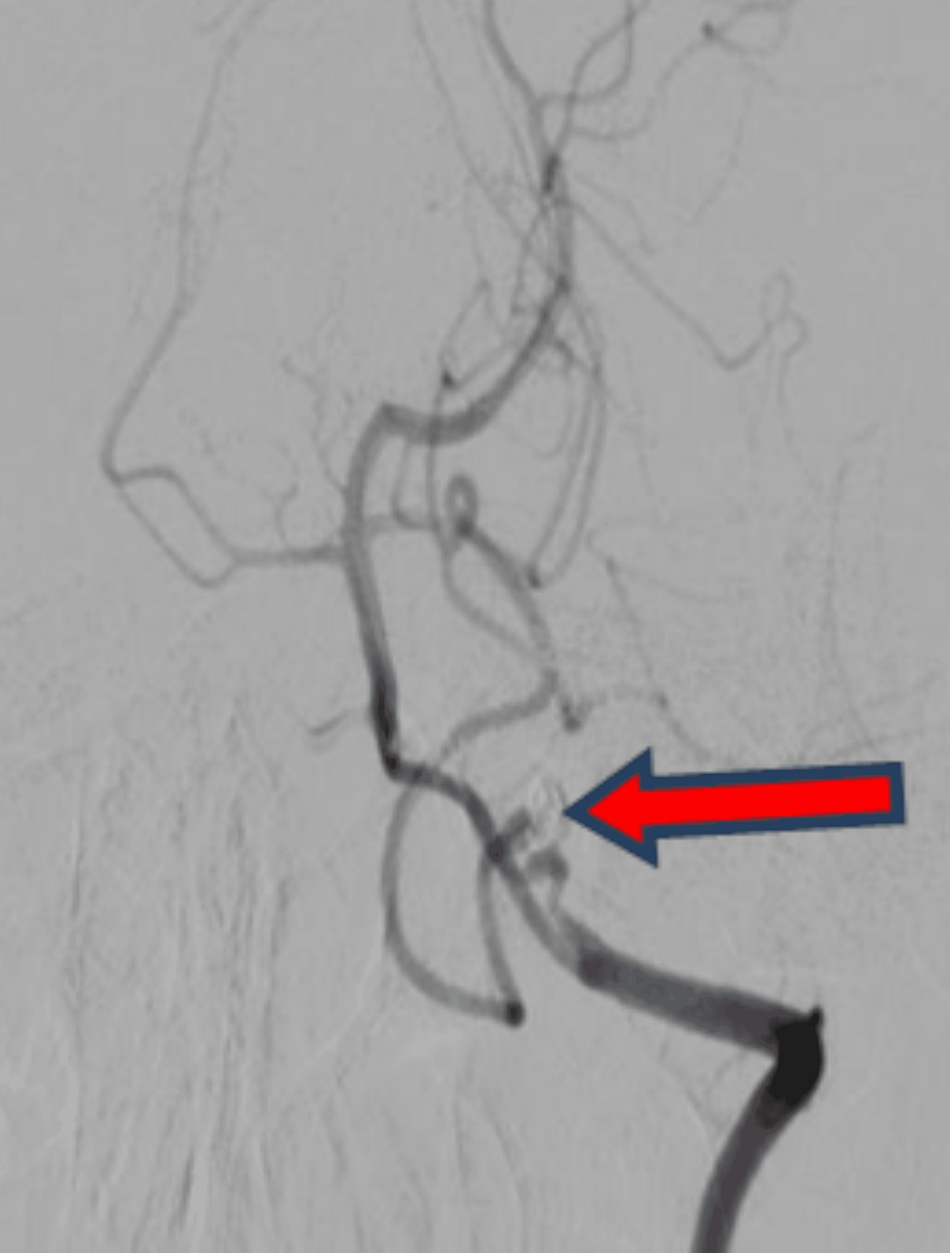
Six months follow up control left vertebral angiogram stable occlusion of left posterior inferior cerebellar artery aneurysm (red arrow) with normal opacification of posterior inferior cerebellar artery and vertebro-basilar circulation.

At follow-up after one month, a computed tomography brain scan was repeated, which was suggestive of mild dilatation of the fourth ventricle. The patient was medically managed. At the six-month follow-up, a cerebral angiogram was repeated, which showed "stable occlusion without any re-rupture or bleed, as shown below in Figure 7."

## Discussion

Aneurysms of the PICA are rare, comprising approximately 0.5-3% of all intracranial aneurysms [2]. Patients with ruptured PICA aneurysms show worse outcomes when compared to aneurysms in other locations.

The PICA has variable anatomy in both its origin and tortuous course, from the vertebral artery and through the lower cranial nerves, and varied treatment options depending on patency and relationship with the vertebral artery. Surgery for these aneurysms has been a major challenge because of their location beneath and very close to vital structures like the medulla and cranial nerves IX, X, and XI [4].

The PICA is divided into five segments: anterior medullary (P1), lateral medullary (P2), tonsillomedullary (P3), telovelotonsillar (P4), and cortical (P5).

True aneurysms could arise from any of the five segments mentioned above. The most common origin is the vertebral artery-PICA junction. Proximal aneurysms are those located at the VA-PICA junction and on segments P1-P3. P4 and P5 aneurysms are distal aneurysms. Proximal aneurysms and distal aneurysms share different surgical strategies. A complex aneurysm that cannot be treated by simple coiling or clipping might have complex features: giant (≥25 mm) or large (10-25 mm) size, atherosclerosis, dissecting or fusiform morphology, wide neck, and recurrence after previous coiling or clipping [5].

PICA patients are mostly prone to persistent lower cranial nerve dysfunction (cough, hoarseness of voice, etc.). Post-operative complications commonly observed are lateral medullary syndrome and aspiration pneumonia secondary to lower cranial nerve palsies.

Patients are pre-operatively checked with either conventional digital subtraction angiography or computed tomography angiography. Both microsurgical and endovascular treatments (EVTs) are done. However, long-term data are lacking [6]. PICA aneurysms with complex features require immediate surgical intervention. Dissecting and/or distal PICA aneurysms were treated with clip wrapping, clip reconstruction, or aneurysm trapping with bypass, as determined by the interventionist. Patients were surgically managed with primary coil occlusions without stent placement. [7]

Neurosurgical clipping while preserving PICA is challenging because of its deep anatomical location and the significant risk of complications. Hence, EVT via coiling has been preferred over neurosurgical clipping in recent times. In recent years, for ruptured aneurysms, coil embolization has been the treatment of choice. Endovascular therapy should be considered first-line therapy for ruptured PICA aneurysms. Coil embolization is not easy in ruptured PICA aneurysms due to variable anatomy and less expertise in its relationship to the parent artery. Surgical clipping and endovascular coiling are effective in preventing re-bleeding and should be preferred as early as possible. In the acute phase, surgical approaches in the proximal PICA have high risk, and post-operative neurological deficits are seen in ≥50% of the cases. It is quite common to encounter complications of coiling, such as aneurysm rupture or thromboembolism of coiling [8]. A very high rate of procedural rupture occurred in PICA aneurysms treated with coil occlusion. Preservation of the parent vessel was achieved by microcatheter auto-assistance without using a balloon or stent. Stent-assisted coiling has the risk of rebleeding but restores circulation. Therefore, microsurgical treatment may be required for the treatment of complex PICA aneurysms [9].

## Conclusions

Headache and vertigo can be predictors of life-threatening conditions like the rupture of an aneurysm, but if diagnosed on time, they can be managed effectively. Endovascular therapy should be considered the first-line and most appropriate treatment with a good functional outcome for ruptured PICA aneurysms. Here, we described a young patient, a 31-year-old female, who presented with headache and vertigo with a ruptured complex PICA aneurysm. The patient received a prompt diagnosis and successfully underwent primary coiling treatment. The patient is on regular follow-up and has satisfactory progress.
